# A comprehensive analysis of Aurora A; transcript levels are the most reliable in association with proliferation and prognosis in breast cancer

**DOI:** 10.1186/1471-2407-13-217

**Published:** 2013-04-30

**Authors:** Satoko Yamamoto, Mutsuko Yamamoto-Ibusuki, Yutaka Yamamoto, Saori Fujiwara, Hirotaka Iwase

**Affiliations:** 1 Department of Breast and Endocrine Surgery, Graduate School of Medical Sciences, Kumamoto University, 1-1-1 Honjo Kumamoto, Kumamoto 860-8556, Japan; 2 Department of Molecular-Targeting Therapy for Breast Cancer, Innovation Center for Translational Research, Kumamoto University Hospital, 1-1-1 Honjo Kumamoto, Kumamoto 860-8556, Japan

**Keywords:** Aurora A, Breast cancer, Biomarker, Transcript levels

## Abstract

**Background:**

Aurora A kinase, a centrosomal serine/threonine kinase which plays an essential role in chromosome segregation during cell division, is commonly amplified and/or over expressed in human malignancies. Aurora A is suggested to be one of the proliferation parameters which is an independent prognostic factor for early invasive breast cancer patients; however the individual clinical or prognostic relevance of this gene has been a matter of debate.

**Methods:**

A comprehensive analysis of Aurora A at the levels of gene expression, gene copy number and protein expression was performed for 278 primary invasive breast cancer patients; and the correlation with clinical outcomes were investigated.

**Results:**

Aurora A gene expression level not only correlated with gene amplification, but was also significantly associated with several clinicopathological parameters and patient prognosis. Patients with higher nuclear grade, negative progesterone receptor status and higher Ki67 expressed higher levels of Aurora A mRNA, which was associated not only with poor relapse-free survival (RFS) but was also found to be a significant multivariate parameter for RFS. Aurora A protein expression was also significantly associated with clinicopathological characteristics; lymph node status, nuclear grade, estrogen receptor status and Ki67, but not with prognosis. By contrast, Aurora A gene amplification correlated with tumor size, nuclear grade and Ki67, and had no prognostic value.

**Conclusion:**

Our data indicate that Aurora A gene expression is an effective tool, which defines both tumor proliferation potency and patient prognosis.

## Background

Aurora kinases, centrosomal serine/threonine kinases, are members of the kinase family involved in cell division, and play an essential role in chromosome segregation during cell division through their establishment of bipolar spindles. There are three types of Aurora kinases in mammals, Aurora A, B and C. They differ in length and in the sequence of the amino terminal domain and have different intracellular locations reflecting their different functions in the cell cycle [1]. The human Aurora A gene is located on chromosome segment 20q13, a segment which is commonly amplified and/or overexpressed in several human epithelial malignancies, including colon, bladder, ovary, pancreas, and breast [2-6]. Aurora A amplification and/or overexpression has been associated with centrosome anomalies and chromosomal instability as well as abrogation of DNA damage-induced apoptotic response and spindle assembly checkpoint override in tumor cells, and as a result Aurora A was defined as an oncogene [7,8]. Furthermore, Aurora A overexpression has been found to correlate with phosphorylation of tumor suppressors such as p53, thereby modulating their activities [9] and suggesting a role in the unregulated proliferation and resistance to DNA damage-induced apoptosis in breast cancer [10].

Nowadays, intrinsic molecular subtypes identified by global microarray-based gene expression analysis can be used to categorize breast cancer, which displays great diversity and molecular heterogeneity [11]. Moreover, more convenient tools for the analysis of gene expression together with clinical outcome data, such as Oncotype Dx [12], Mammaprint [13], and PAM50 [14], have been developed and used in prognostic assessments and prediction of therapeutic efficacy for high risk of recurrence in early breast cancer patients. These gene analysis tools include the majority of proliferation or cell-cycle-related genes, including Aurora A, which acts as a powerful prognostic factor in line with estrogen receptor (ER) or human epidermal growth factor receptor type2 (HER2) status. In view of the clinical significance of Aurora A in breast cancer, overexpression has been correlated with high nuclear grade in only a tiny number of studies but these studies indicate that gene amplification and/or overexpression of Aurora A are linked to tumorigenesis [1,2,7]. In particular, a recent study suggested that Aurora A protein expression outperforms other proliferation makers, such as Ki67 protein, in ER positive breast cancer [15]. Whereas several studies have assessed the expression levels of Aurora A itself, exploring its clinical significance, there have been none which have compared mRNA expression, copy number aberration and protein expression, and the correlation of each.

In the present study, we examined the expression levels of Aurora A (*AURKA*) mRNA, amplification of gene copy number and protein expression in a cohort of patients with primary invasive breast cancer. The relationship between Aurora A status and clinicopathological characteristics and prognosis was evaluated.

## Methods

### Patients and breast cancer tissues

Breast tumor specimens from 278 consecutive female patients with primary invasive breast carcinoma**,** who were treated at Kumamoto University Hospital between 2001 and 2008**,** were included in this study. No exclusion criteria were applied. The study was reported according to the Reporting Recommendations for Tumor Marker Prognostic Studies (REMARK) criteria [16]. All patients had undergone pretherapeutic biopsy or surgical treatment. Samples were snap frozen in liquid nitrogen and stored at −80°C until used for simultaneous total RNA and genomic DNA extraction. Adjuvant treatment and neoadjuvant treatment were decided by risk evaluation according to tumor biology (ER, PgR, and HER2 except Ki-67 status) and clinical staging, including sentinel lymph node biopsy, in accordance with the recommendations of the St. Gallen international expert consensus on the primary therapy of early breast cancer [11,17-19]. In detail, neoadjuvant treatments were administered to 62 patients; 46 of whom received chemotherapy and 16, hormonal therapy. The breast conserving rate was 68.2%, and most of these were treated with radiotherapy. Axillary lymph node dissection was carried out in 45.2% of cases; others were omitted dissection due to negative lymph node status by sentinel node exploration. A total of 208 patients were treated with hormone therapy; aromatase inhibitors (AI): 124, tamoxifen (TAM): 20, TAM-AI: 20, ovarian function suppression plus TAM: 44. One hundred six patients were administered chemotherapy; anthracycline-containing regimens (ACR) followed by taxanes: 68, ACR only: 20, taxanes only: 9, others: 9, and 19 patients were treated with trastuzumab. The ethics committee of Kumamoto University Graduate School of Medical Sciences approved the study protocol. Informed consent was obtained from all patients. Patients were followed postoperatively every 3 months. The median follow-up period was 53 months (range 5-121 months).

### RNA extraction and real-time quantitative reverse transcription-polymerase chain reaction

Total RNA was isolated from the 278 snap-frozen specimens using an RNeasy Mini Kit (Qiagen, Germantown, MD, USA) according to the manufacturer’s instructions. RNA was quantified by measuring the A260/A280 absorbance ratios (Nano-Drop Technologies, Wilmington, DE). RNA was qualitatively assessed using the Agilent 2100 Bioanalyzer (Expert Software version B.02.03) with RNA Nano LabChip Kits (Agilent Technologies, Stockport, UK). Total RNA (0.5 μg) was reverse transcribed to cDNA using PrimeScript® RT Master Mix (Takara Bio Inc., Otsu, Japan), according to the manufacturer's protocol. Reverse transcription real-time quantitative polymerase chain reaction (RT-qPCR) was performed with 15 ng of the cDNA and 0.2 μmol/L of each assay in the ABI Prism 7500 (Applied Biosystems, Carlsbad, CA) by the comparative method with TaqMan chemistry. PCR primers were as follows: TaqMan gene expression assay *AURKA*; Hs01582073_m1, *ACTB;* Hs01060665_g1, *PUM1;* Hs00982775_m1, *TAF-10;* Hs00359540_g1 (Applied Biosystems). Each reaction was performed under the following conditions: initialization for 20 s at 95°C, and then 40-cycles of amplification, comprising 3 s at 95°C for denaturation and 30 s at 60°C for annealing and elongation. The maximum cycle threshold (Ct) value was set at 40. Relative expression values of each gene per sample (the raw Ct data) were calculated by SDS 2.2 software (Applied Biosystems), with expression defined as the point at which the fluorescence rises above the background fluorescence. Data Assist® software (Applied Biosystems) was used to calculate relative gene expression by the delta-Ct method normalized with our in-house multiple reference genes.

### Gene copy number

Patient and control genomic DNA was extracted using the Allprep DNA/RNA Mini Kit (Qiagen) following the manufacturer’s protocol. The concentration and purity of the genomic DNA preparations were measured. Aurora A gene amplification was analyzed with copy number assay by RT-qPCR on a PRIZM 7500 real-time PCR System (Applied Biosystems, Foster city, CA). RNase P was chosen as a reference for gene dosage because of its single copy number. Each reaction was performed in triplicate in a total volume of 20 μL, including 4 μL of gDNA, 1 μL of *AURKA* TaqMan Copy Number Assay (Hs02052288_cn, Applied Biosystems), 1 μL of RNase P TaqMan Copy Number Reference Assay (4316844, Applied Biosystems), and 10 μL of Master Mix. Thermal cycling conditions included an initialization step at 95°C for 10 min, followed by 40-cycles of 15 s at 95°C and 60 s at 60°C. Calculation of the gene copy number was carried out using the absolute quantification method. Aurora A gene status was defined by the ratio of *AURKA* versus RNase P gene. The cut-off level was investigated with 40 cases of normal breast tissue (Additional file 1: Figure S1), which defined a ratio of 1.70, the upper limit of 95% confidential interval, indicating amplification.

### Immunohistochemistry and scoring system

Histological sections (4 μm) were deparaffinized and incubated for 10 min in methanol containing 0.3% hydrogen peroxide. We used rabbit polyclonal antibody against Aurora A (Histofine MAX-PO, 1:100, Nichirei, Japan), which targeted the N terminal of Aurora A kinase. We also used mouse monoclonal antibodies against ERα (SP1, Ventana Japan, Tokyo, Japan), progesterone receptor (PgR) (1E2, Ventana Japan) and Ki67 (MIB1, Dako Japan, Tokyo, Japan), and a polyclonal antibody against Her2 (Dako Japan, 1:200); staining was carried out in the NexES IHC Immunostainer (Ventana Medical Systems, Tucson, AZ), in accordance with the manufacturer's instructions. Aurora A expression was scored according to the respective different staining patterns, predominantly cytoplasmic, however nuclear staining was also seen. We evaluated each pattern of staining and further combined scoring, which turned the cytoplasmic staining out to be mostly correlated with clinical information. Thus we scored the percentage of cytoplasmic staining in the positively-stained tumor cells, as the same way with Royce ME et al. [20]. Specimens in which >50% of cells were stained were scored as strongly positive (3+), those in which >20-50% of cells were stained were scored as moderately positive (2+), those in which >5-20% of cells were stained were scored as weakly positive (1+), and those in which <5% of cells were stained, or where there was no staining, were scored as negative (0). Ki67 was scored as the percentage of nuclear-stained cells out of all cancer cells along the invasive front of the tumor in ×400 high-power fields; this gave the Ki67 labeling index. ER and PgR status were evaluated based on the percentage of positively-stained nuclei and the status of each was considered positive when there was ≥1% of nuclear staining [21]. Her2 was evaluated using the HercepTest method (Dako), with membranous staining scored on a scale of 0 to 3+. Tumors with scores of ≥3 or with a ≥2.2-fold increase in HER2 gene amplification as determined by fluorescence *in situ* hybridization were considered to be positive for Her2 overexpression.

### Statistical analysis

The nonparametric Wilcoxon (for uni-variable), Kruskal-Wallis test (for multi-variables), and the χ^2^ test was adopted for statistical analysis of the associations between different Aurora A status and clinicopathological factors. Relapse-free survival (RFS) and breast cancer-specific survival (BCSS) curves were calculated according to the Kaplan-Meier method and verified by the log-rank test. Univariate and multivariate analyses of prognostic values were performed with the Cox’s proportional hazards model. All statistical significance was defined as *P* < 0.05. JMP software version 8.0.2 for Windows (SAS institute Japan, Tokyo, Japan) was used for all statistical analyses.

## Results

### Correlation between Aurora A mRNA expression, gene copy number and protein expression

**Figure 1:**
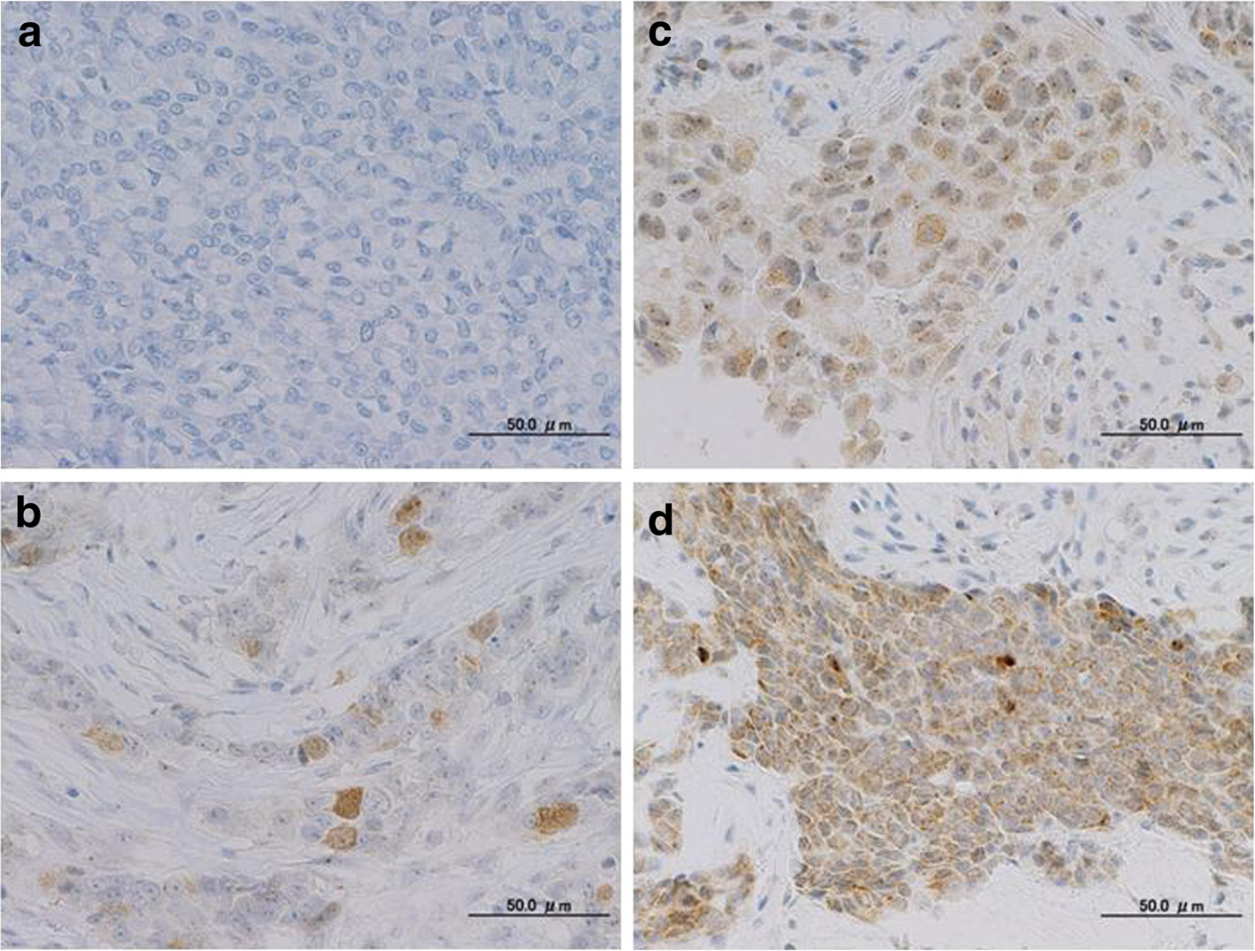
Immunohistochemical staining patterns of Aurora A: a negative staining (no staining, <5%); b weakly positive (≤5%, <20%); c moderately positive (≥20%, <50%); d strongly positive (≥50%). (Magnification ×400).

We analyzed Aurora A mRNA expression, gene copy number and protein expression in 278 primary invasive breast tumors. Relative Aurora A mRNA expression ranged from 0.0001 to 45.36 (median, 0.164). The median value for the ratio of Aurora A versus RNase P was 1.52. In total, 63 (23%) cases showed a ratio <1.0, 174 (63%) cases showed a ratio from 1.0 to 2.0, 37 (13%) cases were from 2.0 to 4.0, and 4 (1%) cases were >4.0. If a ratio >1.70 is defined as positive amplification, 78 (28%) cases were positive and 200 (72%) cases were negative. Representative staining patterns for each Aurora A protein expression level are shown in Figure 1. Sixty-eight (24%) cases were weakly positive: 1+, moderate: 2+, and strong: 3+ expression was present in 38 (14%), 13 (5%), and 17 (6%) cases, respectively, and 210 (75%) cases were negative: 0.

When we dichotomized Aurora A gene copy number into positive and negative, Aurora A mRNA level was higher in the patients exhibiting gene amplification; patients were divided into groups of positive (n=78; median mRNA; 0.209) and negative expression (n=200; median mRNA; 0.157) both in the entire cohort (*P* = 0.017; Additional file 1: Figure S2a) and in the ER+/HER2- subtype group (*P* = 0.0035; Additional file 1: Figure S2b), but not in the ER+ or- /HER2+ subtype group or the ER-/HER2- (triple-negative) subtype group. Furthermore, dividing into four subgroups according to the protein expression levels (negative, weakly positive, moderately positive and strongly positive groups); Aurora A mRNA median levels were 0.157, 0.228, 0.067 and 0.578, respectively (Additional file 1: Figure S3). There was mild correlation between Aurora A mRNA and protein expression in the entire cohort (*P* = 0.075), but no correlation within each subtype. Additionally, no significant correlation was indicated between Aurora A amplification and protein expression in the entire cohort (*P* = 0.553; Additional file 2: Table S1).

### Association of Aurora A mRNA expression, gene copy number and protein expression with clinicopathological characteristics

We examined the relationship between Aurora A mRNA expression, gene copy number and protein expression, and clinicopathological characteristics (Table 1). The level of Aurora A mRNA expression was significantly associated with several clinicopathological parameters. Higher Aurora A mRNA levels were seen in the group of patients with higher nuclear grade (*P* = 0.0004), negative PgR status (*P* = 0.016), as well as higher Ki67 labeling index (*P* < 0.0001). No significant relationship could be found between the subtype groups, but the triple negative group showed the highest Aurora A mRNA expression levels among all subtypes.

**Table 1:** Association of Aurora A mRNA expression, gene copy number and protein expression with clinicopathological parameters

**Clinical parameters**	**No. of patients**	**Aurora A mRNA expression**		**Aurora A amplification**					**Aurora A protein expression**				
		**Median(25%, 75%)**	***P***	**Negative(%)**		**Positive (%)**		***P***	**Negative(%)**		**Positive(%)**		***P***
Age(years)													
<50	65	0.20 (0.08, 0.46)	0.67^*^	42	(65)	23	(35)	0.14^‡^	47	(72)	18	(28)	0.49^‡^
≥50	213	0.16 (0.07, 0.43)		158	(74)	55	(26)		163	(77)	50	(23)	
Menopause													
Pre-	73	0.18 (0.07, 0.42)	0.89^*^	50	(68)	23	(32)	0.45^‡^	54	(74)	19	(26)	0.72^‡^
Post-	205	0.16 (0.08, 0.45)		150	(73)	55	(27)		156	(76)	49	(24)	
Tumor size (mm)													
≤20	124	0.16 (0.08 , 0.36)	0.25^*^	97	(78)	27	(22)	0.035^‡^	99	(80)	25	(20)	0.13^‡^
>20	154	0.18 (0.08, 0.52)		103	(67)	51	(33)		111	(72)	43	(28)	
Nodal status													
-	165	0.16 (0.06, 0.38)	0.14^*^	125	(76)	40	(24)	0.089^‡^	134	(81)	31	(19)	0.0083^‡^
+	113	0.17 (0.09, 0.52)		75	(66)	38	(34)		76	(67)	37	(33)	
Nuclear grade													
1	143	0.14 (0.06, 0.27)	0.0004^†^	114	(80)	29	(20)	0.0044^‡^	123	(86)	20	(14)	<0.0001^‡^
2	66	0.22 (0.10, 0.57)		38	(58)	28	(42)		45	(68)	21	(32)	
3	68	0.31 (0.10, 0.76)		48	(71)	20	(29)		41	(60)	27	(40)	
ER													
-	61	0.20 (0.09, 0.66)	0.14^*^	43	(70)	18	(30)	0.78^‡^	38	(62)	23	(38)	0.0084^‡^
+	217	0.16 (0.08, 0.40)		157	(72)	60	(28)		172	(79)	45	(21)	
PgR													
-	94	0.23 (0.10, 0.56)	0.016^*^	71	(76)	23	(24)	0.34^‡^	67	(71)	27	(29)	0.24^‡^
+	184	0.14 (0.06, 0.38)		129	(70)	55	(30)		143	(78)	41	(22)	
HER2													
-	236	0.16(0.08, 0.42)	0.86^*^	169	(72)	67	(28)	0.77^‡^	183	(76)	53	(22)	0.075^‡^
+	42	0.15(0.08, 0.48)		31	(74)	11	(26)		27	(64)	15	(36)	
Ki67													
≦15%	91	0.11(0.06, 0.20)	<0.0001^*^	74	(81)	17	(18)	0.013^‡^	79	(87)	12	(13)	0.0015^‡^
> 15%	187	0.25(0.10, 0.58)		126	(67)	61	(32)		131	(70)	56	(30)	
Tumor Subtype													
ER+/HER2-	205	0.16(0.07, 0.39)	0.084^†^	150	(73)	55	(27)	0.39^‡^	162	(79)	43	(21)	0.082^‡^
ER+ or –/HER2+	42	0.15(0.08, 0.48)		31	(74)	11	(26)		27	(64)	15	(36)	
ER-/HER2- (Triple Negative)	31	0.29(0.10, 0.79)		19	(61)	12	(39)		21	(68)	10	(32)	

Statistical methods; ^*^:Wilcoxon test, ^†^:Kruskal-Wallis test, ^‡^χ^2^-test.

The Aurora A gene copy number had a less remarkable relationship with clinicopathological factors. Positive amplification was associated with larger tumor size (*P* = 0.035), intermediate nuclear grade (*P* = 0.0044), and higher Ki67 labeling index (*P* = 0.013). Aurora A protein expression was associated with several clinicopathological parameters, as well as Aurora A mRNA, such as positive nodal status (*P* = 0.0083), higher nuclear grade (*P* < 0.0001), negative ER status (*P* = 0.0084), and higher Ki67 labeling index (*P* = 0.0015).

In the ER+/HER2- subtype group (n = 205), higher Aurora A mRNA expression was associated with higher nuclear grade (*P* = 0.0078) and higher Ki67 labeling index (*P* = 0.0005). Positive amplification was associated with higher tumor size (*P* = 0.0051), nuclear grade 2 (*P* = 0.0080) and higher Ki67 labeling index (*P* = 0.0030). Aurora A protein expression was associated with positive nodal status (*P* = 0.018), higher nuclear grade (*P* = 0.0020) and higher Ki67 labeling index (*P* =0.0030; Additional file 2: Table S2). However, in the ER+ or- /HER2+ subtype group (n = 42) and the ER-/HER2- (triple-negative) subtype group (n = 31), neither Aurora A mRNA level, gene amplification nor protein expression showed any significant association with clinicopathological parameters except that Aurora A mRNA expression was higher with higher Ki67 labeling index in the triple negative group (*P* = 0.0026; Additional file 2: Tables S3-4).

### Prognostic relevance of Aurora A mRNA expression, gene copy number and protein expression

In the analysis of RFS, both local recurrences and distant metastases were considered as events. Among 31 recurrent cases, there were 25 cases of distant metastases and 6 of local recurrences. Twenty patients died as a result of breast cancer, and these were regarded as events when analyzing BCSS. The prognostic relevance of Aurora A mRNA, gene copy number and protein expression are summarized in Tables 2 and 3. Our data indicate that Aurora A mRNA expression is an independent predictive factor of a poor prognosis in RFS for primary invasive breast cancer, and is especially superior to Ki67. In the Cox’s proportional hazards model, which included age, menopausal status, tumor size, nodal status, nuclear grade, ER, PgR, HER2 and Ki67, Aurora A mRNA expression proved to be a significant prognostic univariate parameter (*P* = 0.006) and multivariate factor (*P* = 0.027) for RFS (Table 2). As for BCSS, Aurora A mRNA expression was not a significant univariate parameter (*P* = 0.14; Table 3).

**Table 2:** Univariate and multivariate analysis for relapse-free survival (Cox’s proportional hazards model)

**Variable**	**Univariate analysis**			**Multivariate analysis**		
	**HR**	**95% CI**	***P***	**HR**	**95% CI**	***P***
Age	1.03	0.46–2.59	0.95			
Menopausal status	0.89	0.42–2.04	0.77			
Tumor size	2.97	1.35–7.46	0.006	2.51	1.05–6.95	0.037
Nodal status	2.07	1.02–4.31	0.044	1.81	0.86–3.96	0.12
Nuclear grade	1.94	1.28–3.01	0.002	1.1	0.63–1.93	0.74
ER	0.21	0.11–0.44	<0.0001	0.29	0.12–0.72	0.0078
PgR	0.32	0.15–0.65	0.0017			
Her2	1.26	0.47–2.87	0.63			
Ki67 Labeling Index	2.3	0.96–6.82	0.063			
Aurora A mRNA expression	2.54	1.25–5.25	0.010	2.3	1.10–4.92	0.027
Aurora A amplification	1.32	0.60–2.75	0.47			
Aurora A protein expression	1.45	0.65–3.01	0.35			

**Table 3:** Univariate and multivariate analysis for breast cancer specific survival (Cox’s proportional hazards model)

**Variable**	**Univariate analysis**			**Multivariate analysis**		
	**HR**	**95% CI**	***P***	**HR**	**95% CI**	***P***
Age	1.73	0.58–7.40	0.35			
Menopausal status	1.2	0.46–3.71	0.72			
Tumor size	4.64	1.56–19.90	0.0042	3.56	0.92–23.44	0.068
Nodal status	2.64	1.08–7.02	0.033	2.35	0.90–6.92	0.083
Nuclear grade	2.08	1.22–3.68	0.0073	0.94	0.48–1.91	0.86
ER	0.1	0.04–0.26	<0.0001	0.13	0.04–0.42	0.0005
PgR	0.14	0.04–0.38	<0.0001			
Her2	0.88	0.21–2.63	0.84			
Ki67 Labeling Index	3.74	1.07–23.59	0.037	1.6	0.43–10.38	0.52
Aurora A mRNA expression	1.94	0.79–4.76	0.14			
Aurora A amplification	0.86	0.28–2.24	0.78			
Aurora A protein expression	1.38	0.49–3.47	0.52			

**Figure 2:**
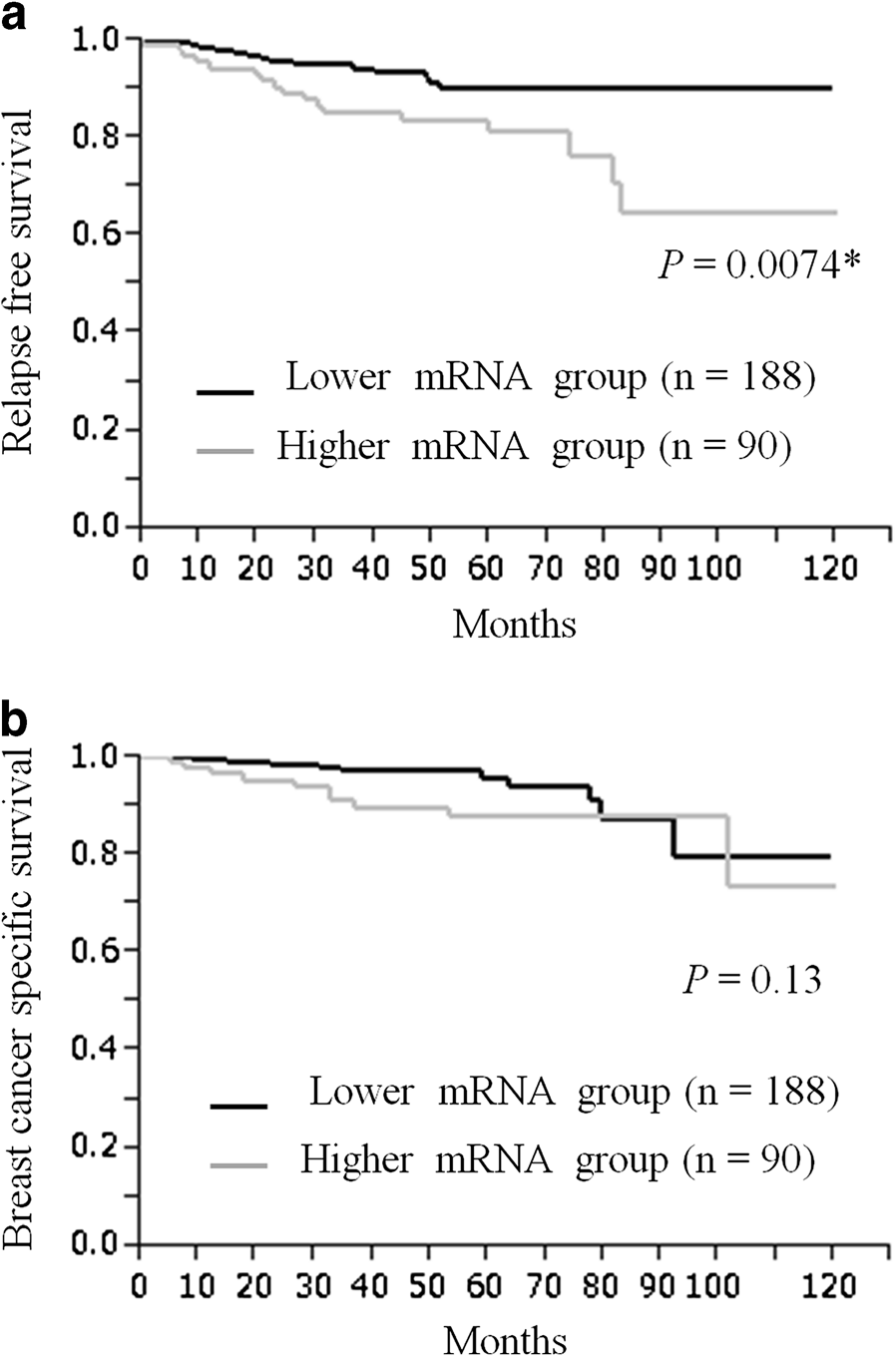
**Aurora A mRNA expression and survival. **Kaplan-Meier plots of the association of Aurora A mRNA expression with relapse-free survival (RFS) (**a**) and breast cancer-specific survival (BCSS) (**b**) in the entire cohort.

To identify a clinically meaningful cut-off for Aurora A mRNA expression, various levels of Aurora A mRNA expression were tested by the Kaplan-Meier method and verified by the log-rank test. An Aurora A mRNA expression level of 0.30 was identified as providing the most significant association with RFS. In this setting, patients with high expression levels (n = 90, median 0.684) had significantly poorer RFS than those with low expression levels (n = 188, median 0.101) (*P* = 0.0074; Figure 2a).

**Figure 3:**
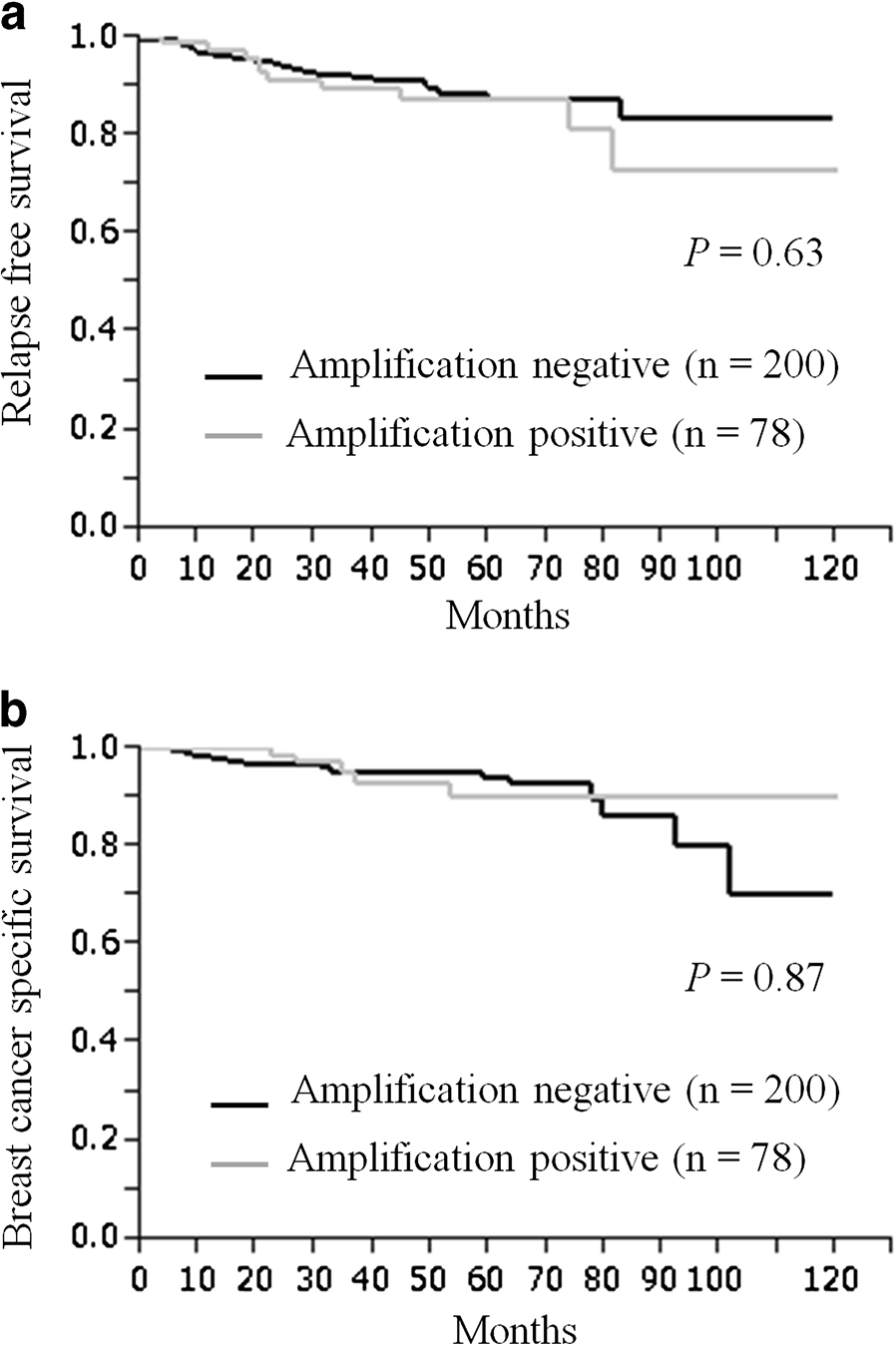
**Aurora A amplification and survival.** Kaplan-Meier plots of the association of Aurora A amplification with relapse-free survival (RFS) (**a**) and breast cancer-specific survival (BCSS) (**b**) in the entire cohort.

**Figure 4:**
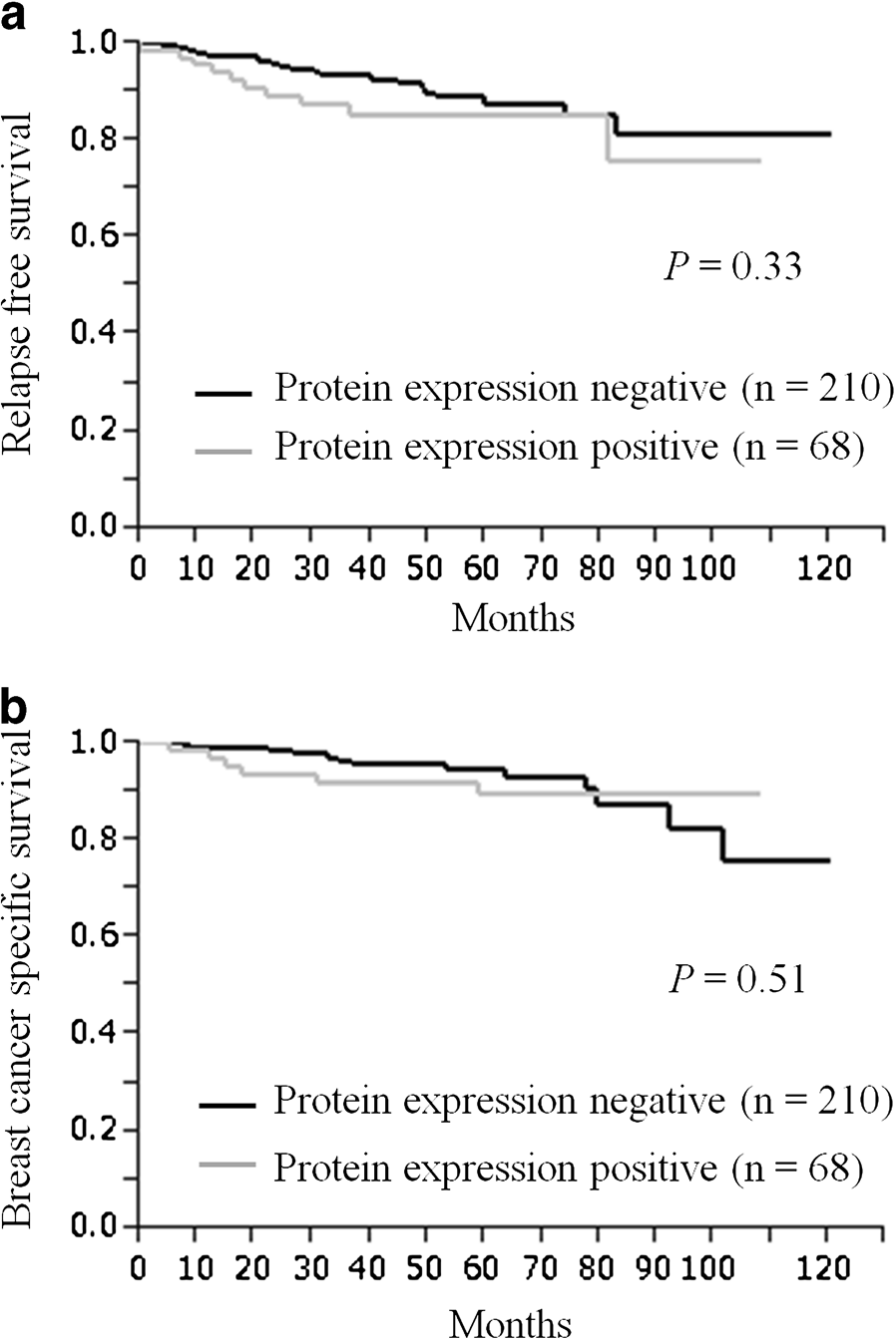
**Aurora A protein expression and survival.** Kaplan-Meier plots of the association of Aurora A protein expression with relapse-free survival (RFS) (**a**) and breast cancer-specific survival (BCSS) (**b**) in the entire cohort.

Furthermore, we studied the prognostic value of Aurora A in different subtypes of our cohort. In the ER+/HER2- subtype group (n = 205), Aurora A gene expression, gene amplification and protein expression were not significantly associated with either RFS nor BCSS using the Cox’s proportional hazards model (Additional file 2: Table S5) and could not be verified by the Kaplan-Meier curve (Additional file 2: Table S4). Neither the ER+ or- /HER2+ subtype group (n = 42) nor the ER-/HER2- subtype group (n = 31) were not significantly associated with RFS and BCSS, as well as the ER+/HER2- subtype group. When we defined the Aurora A gene amplification and protein expression as either positive or negative, in contrast to Aurora A mRNA expression, there was no prognostic difference between the Aurora A gene amplification and non-amplification groups, and the Aurora A protein positive and negative groups. Neither had significant correlation with RFS (Figures 3a and 4a) or with BCSS (Figures 3b and 4b). Additionally, in univariate analysis, there was no significant prognostic value for either RFS (Aurora A gene amplification: *P* = 0.47, Aurora A protein expression: *P* = 0.35; Table 2) or BCSS (Aurora A gene amplification: *P* = 0.78, Aurora A protein expression: *P* = 0.52; Table 3).

## Discussion

Using a quantitative real time PCR-based assay, we found that 28% of samples in our group showed Aurora A gene amplification, a finding which was superior to the result of Zhou’s research which showed amplification in 12% of primary breast cancer cell lines [22]. Although current studies suggested that copy number aberrations could contribute to increases in DNA instability and lead to genomic imbalance [23], and that copy number aberration has a profound effect on inter-individual variation in gene expression, Aurora A gene amplification did not have any prognostic relevance in our study. We found a significant correlation between the level of Aurora A mRNA expression and gene amplification in the entire cohort (*P* = 0.017; Additional file 1: Figure S2a) and ER+ /HER2- subtype group (*P* = 0.0035; Additional file 1: Figure S2b). It is reasonable that gene amplification should increase the expression level of Aurora A mRNA [23,24], although some cases exhibiting no amplification expressed high Aurora A mRNA levels. In contrast, the association between amplification and protein overexpression was not absolutely significant in the entire cohort (*P* = 0.553; Additional file 2: Table S1); 31% (n = 21) of protein overexpression cases (n = 68) showed gene amplification. Moreover, the correlation between mRNA and protein expression had no significance (*P* = 0.075; Additional file 1: Figure S3). Discrepancies between gene amplification and mRNA and protein over expression rates were previously reported in small cohorts of several cancers [22]. All of those suggested that Aurora A over expression was regulated not only by gene amplification, but also by other mechanisms such as transcriptional activation and suppression of protein degradation. Kimura *et al.* reported the rapid degradation of AIK1 (Aurora A) after mitotic phase and the presence of destruction box-like sequences in AIK1 (Aurora A), which suggested the involvement of the ubiquitin-proteasome system in its degradation [25]. In normal cells, Aurora A protein levels are controlled at least in part by the Fbxw7 SCF-based E3 ubiquitin ligase [26]. We previously indicated that patients with lower Fbxw7 mRNA expression had a poorer prognosis for BCSS than those with higher expression [27]. In our cohort, for example, the positive Aurora A protein expression group showed lower Fbxw7 mRNA expression than the Aurora A protein negative group (data not shown). Recently, Chen BB et al. reported that Fbxl7, which is also the SCF-based E3 ligase subunit, specifically ubiquitinates and degrades Aurora A to regulate mitotic events in vitro [28]. We speculate Fbxl7 has higher affinity for Aurora A than Fbxw7 which predominantly work during G0 and G1-S phase, and is also of great value for our next investigation. Chen BB et al. also suggest that Aurora A is modulated and regulated by a variety of post-translational modifications, such as phosphorylation, dephosphorylation. The elucidation of these mechanisms and clarifying their clinical significance hasten us for further studies.

We observed that Aurora A mRNA expression was significantly higher in those patients who had active proliferative parameters such as higher nuclear grade, negative hormone receptor and higher Ki67 labeling index, as well as in the protein over expression group. Additionally, Aurora A mRNA expression turned out to be significantly associated with RFS (*P* = 0.0074) by dichotomous Kaplan-Meier curves, as well as the results of univariate and multivariate RFS. Nowadays, gene expression profiling should be considered as an adjunct to high-quality pathology phenotyping which results in an indication of certain therapies for early breast cancer patients [29] that contain Aurora A as one of the proliferation-genes. Heibe-Kains *et al.* reported that mRNA expression predicts classification into four molecular subtypes by quantitative measurement of three genes on an array-based meta-analysis (ESR1, ERBB2 and AURKA) in a large study of breast cancer patients (n = 5715), which identified the major breast cancer intrinsic subtypes and provided robust discrimination for clinical use in a manner very similar to a 50-gene subtype predictor (PAM50) [30]. Our study provided powerful supporting data that coincide with these fundamental investigations using Aurora A mRNA expression data.

Protein over expression of Aurora A in our study was significantly associated with several clinicopathological characteristics such as lymph node status, nuclear grade, hormone receptor expression, and Ki67 labeling index, which is in agreement with other studies demonstrating that Aurora A over expression levels correlated with higher nuclear grade in breast cancer [1,15,20,31]. Ali *et al.* compared the prognostic value of proliferative markers, such as MCM2, Ki67, Aurora A, polo-like kinase 1 (PLK1), GMNN and phosphorylated-histone H3 (PHH3), based on their differential expression in different phases of the cell cycle, then showed that Aurora A is the best prognostic factor, outperforming Ki67 in ER positive breast cancer [15]. Our data also showed that Aurora A protein over expression was associated with proliferation parameters, but no longer had any prognostic significance in the ER+ /HER2- subtype group. One explanation is that Aurora A protein is expressed not only diffusely in the cytoplasm but also as localized staining in the cytoplasm or nucleus. In this study, we analyzed the proportion of tumor cytoplasmic staining based on our previous study [32] , which was still under development to provide universal guidelines as to whether nuclear and cytoplasmic staining or cytoplasmic stainig was positive. Secondly, the immunostaining technique was influenced by the antibody used and the condition of the tissue block. Biopsy tissue, for example, tended to stain strongly, which influenced the studies, and Aurora A positive staining ranged from 15% to 94%. In our tissue, 17 strongly positive cases (6%) were found to contain diffuse staining in the cytoplasma all over the tumor, but in other cases the staining was mainly localized in a part of the tumor. By establishing standardized guidelines for immunohistochemistry, Aurora A may prove to be a more effective proliferation biomarker for select cases which need more intensive cytotoxic therapy.

Recently, several inhibitors of Aurora kinase such as Hesperadin [33], ZM447439 [34], and VX680 [35] have been developed, which have been designed to target the ATP-binding site of Aurora kinases and thus inhibit all three Aurora kinases. *In vivo* and *in vitro* studies show that the inhibition of tumor growth was paralleled by a significant increase in tumor cell apoptosis. Furthermore, VX680 had no effect on the viability of non-cycling primary human cells, probably because the expression and activity of Aurora A kinases is low or undetectable in normal cells, thus molecular inhibitors of Aurora A kinases could be promising anticancer therapeutics [36,37]. Further work is needed to establish patterns of Aurora A expression and the response to Aurora A inhibitors.

## Conclusions

We analyzed Aurora A expression using three different methods, gene expression, copy number variation using RT-qPCR, and protein expression by immunohistochemistry. Aurora A protein expression was associated with aggressive proliferative parameters, whereas Aurora A mRNA expression was associated not only with poor RFS but also turned out to be an independent biomarker for RFS. Our results suggest that Aurora A expression is an effective tool which can detect tumor proliferation potency, and is considered to be an important part of gene expression profiling for the biological diversity of breast cancer. Further research including Aurora A signaling is needed.

## Competing interest

The authors declare that they have no competing interest.

## Authors’ contributions

YY and MI conceived and designed the experiments. SY and MI performed the experiments. SY have contributed to analyze the data and written the paper. MI has been involved in revising the manuscript. SF, SY, MI, YY and HI have contributed for acquisition of clinical data and specimens. YY and HI have given final approval of the version to be published. All authors read and approved the final manuscript.

## Pre-publication history

The pre-publication history for this paper can be accessed here:

http://www.biomedcentral.com/1471-2407/13/217/prepub

## Supplementary Material

Additional file 1: Figure S1 Distribution of the ratio of Aurora A versus RNase P of normal breast tissues. Box plots, where the mean 1.64 were represented by lines, the upper and lower 95% confidential (1.70 and 1.60, respectively); interval by boxes, and the standard errors of ±1.5 by whiskers. **Figure S2.** Correlation between Aurora A mRNA expression and amplification of gene copy number (a) in the entire cohort and (b) in the ER+/HER2- subtype group. **Figure S3.** Correlation between Aurora A mRNA expression and protein expression in the entire cohort. **Figure S4.** Kaplan-Meier plots of the association of Aurora A mRNA expression with RFS (a) and BCSS (b) in the ER+/HER2- subtype group. Click here for file

Additional file 2: Table S1 Correlation between Aurora protein expression and amplification of gene copy number. **Table S2.** Association of Aurora A mRNA expression, gene copy number and protein expression with clinicopathological parameters in the ER+/HER2- subtype group (n = 205). **Table S3.** Association of Aurora A mRNA expression, gene copy number and protein expression with clinicopathological parameters in the ER+ or- /HER2+ subtype group (n = 42). **Table S4.** Association of Aurora A mRNA expression, gene copy number and protein expression with clinicopathological parameters in the ER–/HER2- subtype group (n = 31). **Table S5.** Univariate analysis for relapse free survival and breast cancer specific survival in the ER+/HER2- subtype group (Cox’s proportional hazards model). Click here for file
