# Supplementary figures and images for: A comprehensive analysis of Aurora A; transcript levels are the most reliable in association with proliferation and prognosis in breast cancer
Source: BMC Cancer. 2013 Apr 30;13:217. doi: 10.1186/1471-2407-13-217 (PMC3671980; doi:10.1186/1471-2407-13-217)

**
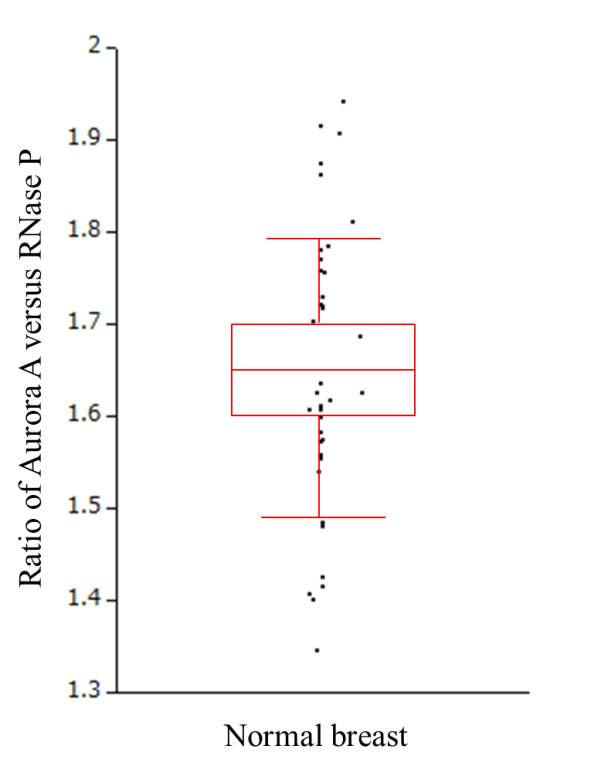
**

**Additional Fig. 1**

**a**


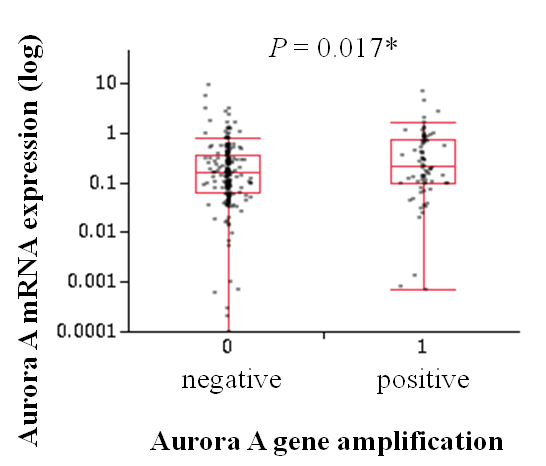


**b**


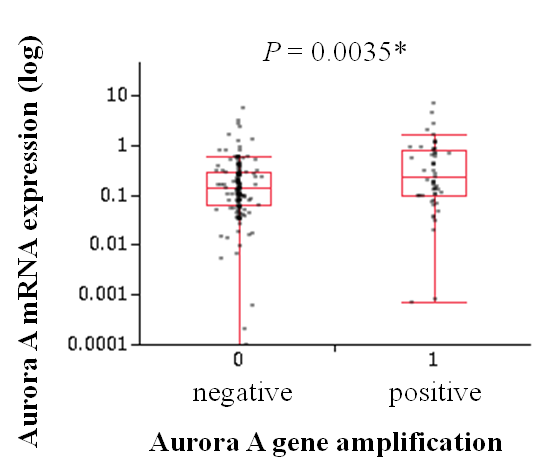


**Additional Fig. 2**

**
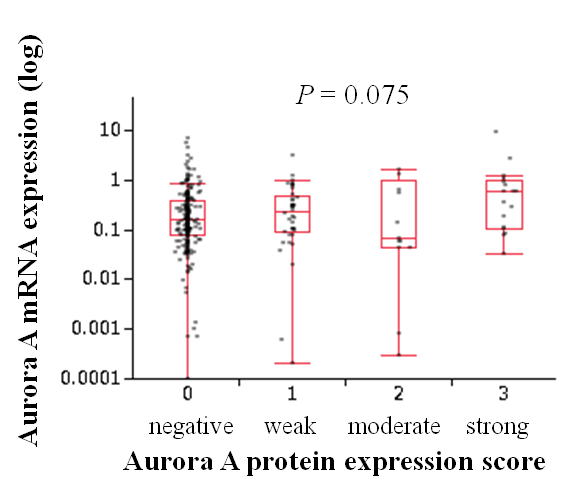
**

**Additional Fig. 3**

**a**


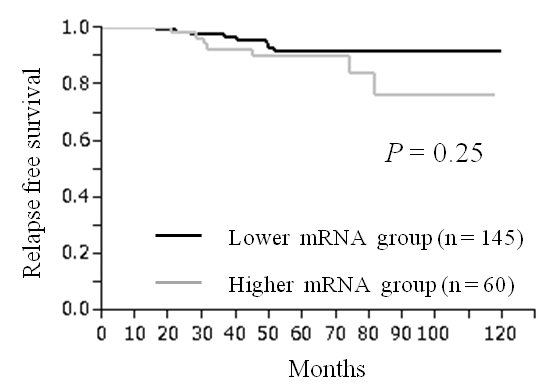


**b**


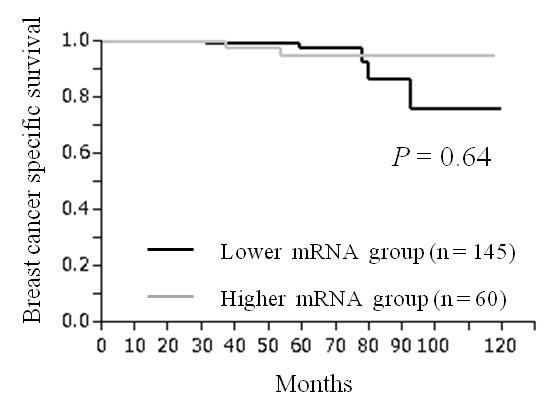


**Additional Fig. 4**

Supplement: Additional file 1: Figure S1 — Distribution of the ratio of Aurora A versus RNase P of normal breast tissues. Box plots, where the mean 1.64 were represented by lines, the upper and lower 95% confidential (1.70 and 1.60, respectively); interval by boxes, and the standard errors of ±1.5 by whiskers. Figure S2. Correlation between Aurora A mRNA expression and amplification of gene copy number (a) in the entire cohort and (b) in the ER+/HER2- subtype group. Figure S3. Correlation between Aurora A mRNA expression and protein expression in the entire cohort. Figure S4. Kaplan-Meier plots of the association of Aurora A mRNA expression with RFS (a) and BCSS (b) in the ER+/HER2- subtype group. [file 1471-2407-13-217-S1.docx]
