# Supplementary material for: A comprehensive analysis of Aurora A; transcript levels are the most reliable in association with proliferation and prognosis in breast cancer
Source: BMC Cancer. 2013 Apr 30;13:217. doi: 10.1186/1471-2407-13-217 (PMC3671980; doi:10.1186/1471-2407-13-217)
Supplement: Additional file 2: Table S1 — Correlation between Aurora protein expression and amplification of gene copy number. Table S2. Association of Aurora A mRNA expression, gene copy number and protein expression with clinicopathological parameters in the ER+/HER2- subtype group (n = 205). Table S3. Association of Aurora A mRNA expression, gene copy number and protein expression with clinicopathological parameters in the ER+ or- /HER2+ subtype group (n = 42). Table S4. Association of Aurora A mRNA expression, gene copy number and protein expression with clinicopathological parameters in the ER–/HER2- subtype group (n = 31). Table S5. Univariate analysis for relapse free survival and breast cancer specific survival in the ER+/HER2- subtype group (Cox’s proportional hazards model). [file 1471-2407-13-217-S2.docx]

**Additional Table 1** Correlation between Aurora protein expression and amplification of gene copy number in the entire cohort.

|  | protein expression | |  |
| --- | --- | --- | --- |
|  | negative | positive |  |
| amplification |  |  |  |
| negative | 153 | 47 | 200 |
| positive | 57 | 21 | 78 |
|  | 210 | 68 | 278 |

*P* = 0.553 (χ^2^ test)

**Additional Table 2** Association of Aurora A mRNA expression, gene copy number and protein expression with clinicopathological parameters in the ER+/HER2-　subtype group (n = 205)

| Clinical parameters | No. of patients |  | AuroraA mRNA expression | |  | AuroraA amplification | | | | |  | AuroraA protein expression | | | | |
| --- | --- | --- | --- | --- | --- | --- | --- | --- | --- | --- | --- | --- | --- | --- | --- | --- |
|  |  |  | Median (25%,75%) | *P* |  | negative(%) | | positive(%) | | *P* |  | negative(%) | | positive(%) | | *P* |
| Age(years) |  |  |  |  |  |  |  |  |  |  |  |  |  |  |  |  |
| <50 | 52 |  | 0.17 (0.08,　0.44) | 0.47 |  | 33 | (63) | 19 | (37) | 0.073 |  | 40 | (77) | 12 | (23) | 0.67 |
| >50 | 153 |  | 0.15 (0.07,　0.36) |  |  | 117 | (76) | 36 | (24) |  |  | 122 | (80) | 31 | (20) |  |
| Menopause |  |  |  |  |  |  |  |  |  |  |  |  |  |  |  |  |
| Pre- | 57 |  | 0.16 (0.07, 0.40) | 0.65 |  | 38 | (67) | 19 | (33) | 0.20 |  | 44 | (77) | 13 | (23) | 0.69 |
| Post- | 148 |  | 0.15 (0.07,　0.37) |  |  | 112 | (76) | 36 | (24) |  |  | 118 | (80) | 30 | (20) |  |
| Tumor size (mm) |  |  |  |  |  |  |  |  |  |  |  |  |  |  |  |  |
| <20 | 96 |  | 0.16 (0.07 ,　0.31) | 0.46 |  | 79 | (82) | 17 | (18) | 0.0051 |  | 80 | (83) | 16 | (17) | 0.15 |
| >20 | 109 |  | 0.16 (0.07,　0.40) |  |  | 71 | (65) | 38 | (35) |  |  | 82 | (75) | 27 | (25) |  |
| Nodal status |  |  |  |  |  |  |  |  |  |  |  |  |  |  |  |  |
| － | 123 |  | 0.14 (0.06,　0.31) | 0.12 |  | 96 | (78) | 27 | (22) | 0.055 |  | 104 | (85) | 19 | (15) | 0.018 |
| ＋ | 82 |  | 0.17 (0.09,　0.46) |  |  | 54 | (66) | 28 | (34) |  |  | 58 | (71) | 24 | (29) |  |
| Nuclear grade |  |  |  |  |  |  |  |  |  |  |  |  |  |  |  |  |
| 1 | 129 |  | 0.14 (0.06,　0.27) | 0.0078 |  | 104 | (81) | 25 | (19) | 0.0080* |  | 112 | (87) | 17 | (13) | 0.0020 |
| 2 | 54 |  | 0.18 (0.09,　0.55) |  |  | 33 | (61) | 21 | (39) |  |  | 35 | (65) | 19 | (35) |  |
| 3 | 22 |  | 0.37 (0.10,　0.61) |  |  | 13 | (59) | 9 | (41) |  |  | 15 | (68) | 7 | (32) |  |
| PgR |  |  |  |  |  |  |  |  |  |  |  |  |  |  |  |  |
| － | 30 |  | 0.24 (0.12,　0.52) | 0.061 |  | 26 | (87) | 4 | (13) | 0.055 |  | 26 | (87) | 4 | (13) | 0.24 |
| ＋ | 175 |  | 0.14 (0.06,　0.38) |  |  | 124 | (71) | 51 | (29) |  |  | 136 | (78) | 39 | (22) |  |
| Ki67(≦15%) |  |  |  |  |  |  |  |  |  |  |  |  |  |  |  |  |
| low | 82 |  | 0.12(0.06,　0.23) | 0.0005 |  | 69 | (84) | 13 | (16) | 0.0030 |  | 73 | (89) | 9 | (11) | 0.003 |
| high | 123 |  | 0.21(0.08,　0.58) |  |  | 81 | (66) | 42 | (34) |  |  | 89 | (72) | 34 | (28) |  |

**Additional Table 3** Association of Aurora A mRNA expression, gene copy number and protein expression with clinicopathological parameters in the ER+ or –/HER2+　subtype group (n = 42)

| Clinical parameters | No. of patients |  | AuroraA mRNA expression | |  | AuroraA amplification | | | | |  | AuroraA protein expression | | | | |
| --- | --- | --- | --- | --- | --- | --- | --- | --- | --- | --- | --- | --- | --- | --- | --- | --- |
|  |  |  | Median(25%,75%) | *P* |  | negative(%) | | positive(%) | | *P* |  | negative(%) | | positive(%) | | *P* |
| Age(years) |  |  |  |  |  |  |  |  |  |  |  |  |  |  |  |  |
| <50 | 9 |  | 0.30 (0.08,　0.49) | 0.89 |  | 6 | (67) | 3 | (33) | 0.59 |  | 5 | (56) | 4 | (44) | 0.54 |
| >50 | 33 |  | 0.14 (0.07,　0.51) |  |  | 25 | (76) | 8 | (24) |  |  | 22 | (67) | 11 | (33) |  |
| Menopause |  |  |  |  |  |  |  |  |  |  |  |  |  |  |  |  |
| Pre- | 12 |  | 0.21 (0.06, 0.51) | 0.96 |  | 9 | (75) | 3 | (25) | 0.91 |  | 8 | (67) | 4 | (33) | 0.84 |
| Post- | 30 |  | 0.15 (0.08, 0.49) |  |  | 22 | (73) | 8 | (27) |  |  | 19 | (63) | 11 | (37) |  |
| Tumor size (mm) |  |  |  |  |  |  |  |  |  |  |  |  |  |  |  |  |
| <20 | 16 |  | 0.14 (0.10 ,　0.54) | 0.88 |  | 11 | (69) | 5 | (31) | 0.56 |  | 13 | (81) | 3 | (19) | 0.06 |
| >20 | 26 |  | 0.18 (0.05,　0.48) |  |  | 20 | (77) | 6 | (23) |  |  | 14 | (54) | 12 | (46) |  |
| Nodal status |  |  |  |  |  |  |  |  |  |  |  |  |  |  |  |  |
| － | 25 |  | 0.18 (0.08,　0.54) | 0.77 |  | 20 | (80) | 5 | (20) | 0.27 |  | 17 | (68) | 8 | (32) | 0.54 |
| ＋ | 17 |  | 0.14 (0.07,　0.38) |  |  | 11 | (65) | 6 | (35) |  |  | 10 | (59) | 7 | (41) |  |
| Nuclear grade |  |  |  |  |  |  |  |  |  |  |  |  |  |  |  |  |
| 1 | 10 |  | 0.13 (0.04,　0.33) | 0.44 |  | 8 | (80) | 2 | (20) | 0.23 |  | 9 | (90) | 1 | (10) | 0.054 |
| 2 | 5 |  | 0.30 (0.13,　0.73) |  |  | 2 | (40) | 3 | (60) |  |  | 4 | (80) | 1 | (20) |  |
| 3 | 27 |  | 0.14 (0.05,　0.56) |  |  | 21 | (78) | 6 | (22) |  |  | 14 | (52) | 13 | (48) |  |
| ER |  |  |  |  |  |  |  |  |  |  |  |  |  |  |  |  |
| － | 28 |  | 0.14 (0.05,　0.51) | 0.75 |  | 23 | (82) | 5 | (18) | 0.088 |  | 17 | (61) | 11 | (39) | 0.49 |
| ＋ | 14 |  | 0.24 (0.10,　0.57) |  |  | 8 | (57) | 6 | (43) |  |  | 10 | (71) | 4 | (29) |  |
| PgR |  |  |  |  |  |  |  |  |  |  |  |  |  |  |  |  |
| － | 33 |  | 0.16 (0.05,　0.49) | 0.99 |  | 26 | (79) | 7 | (21) | 0.18 |  | 20 | (61) | 13 | (39) | 0.33 |
| ＋ | 9 |  | 0.14 (0.09,　0.73) |  |  | 5 | (56) | 4 | (44) |  |  | 7 | (78) | 2 | (22) |  |
| Ki67(≦15%) |  |  |  |  |  |  |  |  |  |  |  |  |  |  |  |  |
| low | 4 |  | 0.10(0.03,　0.39) | 0.37 |  | 3 | (75) | 1 | (25) | 0.95 |  | 3 | (75) | 1 | (25) | 0.63 |
| high | 38 |  | 0.16(0.10,　0.53) |  |  | 28 | (74) | 10 | (26) |  |  | 24 | (63) | 14 | (37) |  |

**Additional Table 4** Association of Aurora A mRNA expression, gene copy number and protein expression with clinicopathological parameters in the ER–/HER2-　subtype group (n = 31)

| Clinical parameters | No. of patients |  | AuroraA mRNA expression | |  | AuroraA amplification | | | | |  | AuroraA protein expression | | | | |
| --- | --- | --- | --- | --- | --- | --- | --- | --- | --- | --- | --- | --- | --- | --- | --- | --- |
|  |  |  | Median (25%,75%) | *P* |  | negative(%) | | positive(%) | | *P* |  | negative(%) | | positive(%) | | *P* |
| Age(years) |  |  |  |  |  |  |  |  |  |  |  |  |  |  |  |  |
| <50 | 4 |  | 0.33 (0.07,　0.47) | 0.48 |  | 3 | (75) | 1 | (25) | 0.54 |  | 2 | (50) | 2 | (50) | 0.43 |
| >50 | 27 |  | 0.28 (0.10,　0.83) |  |  | 16 | (59) | 11 | (41) |  |  | 19 | (70) | 8 | (30) |  |
| Menopause |  |  |  |  |  |  |  |  |  |  |  |  |  |  |  |  |
| Pre- | 4 |  | 0.33 (0.07,　0.47) | 0.48 |  | 3 | (75) | 1 | (25) | 0.54 |  | 2 | (50) | 2 | (50) | 0.43 |
| Post- | 27 |  | 0.28 (0.10,　0.83) |  |  | 16 | (59) | 11 | (41) |  |  | 19 | (70) | 8 | (30) |  |
| Tumor size (mm) |  |  |  |  |  |  |  |  |  |  |  |  |  |  |  |  |
| <20 | 12 |  | 0.18 (0.09 ,　0.66) | 0.19 |  | 7 | (58) | 5 | (42) | 0.79 |  | 6 | (50) | 6 | (50) | 0.09 |
| >20 | 19 |  | 0.52 (0.15,　0.83) |  |  | 12 | (63) | 7 | (37) |  |  | 15 | (79) | 4 | (21) |  |
| Nodal status |  |  |  |  |  |  |  |  |  |  |  |  |  |  |  |  |
| － | 17 |  | 0.22 (0.10,　0.77) | 0.34 |  | 9 | (53) | 8 | (47) | 0.29 |  | 13 | (76) | 4 | (24) | 0.25 |
| ＋ | 14 |  | 0.51 (0.11,　1.10) |  |  | 10 | (71) | 4 | (29) |  |  | 8 | (57) | 6 | (43) |  |
| Nuclear grade |  |  |  |  |  |  |  |  |  |  |  |  |  |  |  |  |
| 1 | 4 |  | 0.11 (0.03,　0.26) | 0.17 |  | 2 | (50) | 2 | (50) | 0.30 |  | 2 | (50) | 2 | (50) | 0.39 |
| 2 | 7 |  | 0.28 (0.12,　1.24) |  |  | 3 | (43) | 4 | (57) |  |  | 6 | (86) | 1 | (14) |  |
| 3 | 19 |  | 0.52 (0.15,　0.83) |  |  | 14 | (74) | 5 | (26) |  |  | 12 | (63) | 7 | (37) |  |
| Ki67(≦15%) |  |  |  |  |  |  |  |  |  |  |  |  |  |  |  |  |
| low | 5 |  | 0.06(0.03,　0.10) | 0.0026* |  | 2 | (40) | 3 | (60) | 0.29 |  | 3 | (100) | 0 | (0) | 0.12 |
| high | 26 |  | 0.51(0.17,　0.87) |  |  | 17 | (65) | 9 | (35) |  |  | 18 | (77) | 6 | (23) |  |

**Additional Table 5** Univariate analysis for relapse-free survival and breast cancer-specific survival in the ER+/HER2- subtype group (Cox’s proportional hazards model)

| variable | Univariate analysis (RFS) | | |  | Univariate analysis (BCSS) | | |
| --- | --- | --- | --- | --- | --- | --- | --- |
|  | HR | 95%CI | *P* |  | HR | 95%CI | *P* |
| Age | 0.87 | 0.29–3.17 | 0.82 |  | 1.59 | 0.27–30.2 | 0.65 |
| Menopausal status | 1.12 | 0.38–4.06 | 0.84 |  | 2.57 | 0.44–48.7 | 0.33 |
| Tumor size | 2.74 | 0.94–9.91 | 0.066 |  | 6.00 | 1.02–113.4 | 0.047* |
| Nodal status | 1.68 | 0.60–4.79 | 0.32 |  | 3.33 | 0.72–23.3 | 0.13 |
| Nuclear grade | 1.45 | 0.71–2.76 | 0.29 |  | 0.86 | 0.22–2.42 | 0.80 |
| PgR | 0.64 | 0.21–2.33 | 0.46 |  | 0.44 | 0.09–2.31 | 0.31 |
| Ki67 Labeling Index | 1.53 | 0.52–5.52 | 0.46 |  | 1.18 | 0.25–8.36 | 0.85 |
| AuroraA mRNA expression | 1.80 | 0.63–5.04 | 0.26 |  | 0.67 | 0.10–3.16 | 0.63 |
| AuroraA amplification | 1.95 | 0.65–5.43 | 0.22 |  | 1.05 | 0.15–4.92 | 0.96 |
| AuroraA protein expression | 1.61 | 0.50–4.54 | 0.40 |  | 1.22 | 0.17–5.70 | 0.81 |
